# Top Shelf Drinks, Bottom Line Play: Examining Representations of Class in Bartending and Mixology Games

**DOI:** 10.1177/15554120221119962

**Published:** 2022-08-10

**Authors:** Scott DeJong, Courtney Blamey

**Affiliations:** 1 Communication, 5618 Concordia University, Montreal, Canada

**Keywords:** video games, bartending, mixology, working-class labor, precarity, service labor, creative labor

## Abstract

There is an emerging body of games that simulate the labor of drink making and serving at the forefront of play through the role of a bartender or artisanal mixologist. Both are working class but the creative variance between them challenges how economic precarity is understood. The authors ask how this translates to video games when these positions are foregrounded. How do play, poverty, and precarity interconnect in drink making and serving games? Through the qualitative analysis of four games that put the player in the position of bartender or mixologist, this paper shows how creative labor and precarity are illuminated or obfuscated through mechanics and narrative. In doing so, it argues how games, as one form of media, obscure or make visible labor and precarity to players and simultaneously reinforce the romanticization of often exploited creative labor. These findings prompt further questions and research directions on representations of working-class labor.

## Introduction

Debuted in arcades, *Tapper* (1983) eats players’ quarters as they control a pixelated bartender to quickly serve customers beers in an attempt to get a high score. As the first in a now a small subset of games focused on placing players at the site of the bar, (Chan, 2019) *Tapper* asked players to be a blue-collar laborer (named Tapper) who races to pour and serve drinks, pick up leftover glasses, and collect tips before the customers get angry enough to throw Tapper out. Since *Tapper,* this burgeoning theme of drink making in games continues to demonstrate how precarity and labor are embedded in play. While labor in *Tapper* is visible, the impacts of social class remain invisible, obfuscated behind play mechanics, interfaces, and narrative discourse.

Across these bartending games, a player's role can often be broken down into two different approaches: the bartender and the mixologist. Tapper emulates a bartender: they pour and serve drinks from kegs and the game focuses on menial but relentless labor. In contrast, the mixologists, seen as artisans and tastemakers, are an experience curator, or cultural intermediary (Ocejo, 2012). These roles usher customers into a distinct moment and atmosphere for consuming alcohol, one suited to individual customer's tastes. The key distinction between the two is creativity, or rather, the value tied to creative labor. Both bartenders and mixologists live on the edge of precarity, and their role as a server or tastemaker masks the larger social class challenges they might face.

Drink making and serving games emphasize creative practice, a fast-paced work environment, and the need to establish a positive customer experience. By studying games where drink making labor is at the forefront of play, we can see how playful spaces further obscure or reflect the relationship between working-class labor and creative practice. From here we study two interrelated research questions: “how is drink making and serving within videogames related to media tropes of social class?” and “how is precarity embedded in videogames through these practises?”

We focus on the theme of drink making and serving games because these activities provide a direct line of comparison for creative and noncreative labor. We explore how games present the “uncreative” bartender compared to the artisanal mixologist to tease out an understanding of how representations of labor can influence a game's understanding of social class. This paper does this through a close analysis of the bartender or mixologist in four games: *Tapper, Bartender the Right Mix, VA-11 Hall-A,* and *Red Strings Club.* Through this comparison, we demonstrate how games can illuminate or obscure the precarity of service labor to argue that drink making games reinforce labor and social class expectations of creativity and precarity.

## Background Literature

### Precarity, Video Games, and Class Labor

Working-class labor within video games is typically offloaded to the supporting cast, elaborated in backstories, or embedded in the world around the character. For many open-world games, this is seen in the roles that non-player characters (NPCs) take, such as the roaming merchants in *Breath of the Wild* (Nintendo of America Inc. 2017) or townsfolk in the *Lost Ark* (Amazon Game Studio and Smilegate 2022) which provide various services. As “heroes”, player characters rarely embody the menial aspects of such labor. This is what makes games focused on working-class labor interesting: labor is almost always successful, and its hardships are removed. Here, the powerful player character is rendered a “working class hero” (Lantorno et al., 2021), someone who is going above and beyond their actual societal position to achieve success while still deeply entrenched in their class position. Often established through stereotypes rather than the labor itself, the author has argued that a player character's class position is typically represented through visual references to tropes, such as a janitorial character holding a broom, rather than a simulation of labor.

For our purposes, working-class labor refers to manual labor. Distinguishing between working and middle class, Butsch (2003) suggests that, “the occupational distinction between middle-class and working-class occupations is primarily that between mental and manual labor” (p. 18). Working-class labor is broad and ranges from minimum wage work, lower management, to trade labor (i.e., a blacksmith in video games). While it is possible for someone to own and bartend their own establishment, alluded to in *Tapper* before the company Budweiser sponsored specific machines, drink making, and serving games typically prioritize a working-class hero who is bound to the will of the establishment or customers. This still places the working class into a broad category, where the wages for different manual labor jobs exist on a wide economic spectrum ranging from relative comfort to perpetual precarity (Draut, 2018). Where might precarity exist (or not) in drink making and serving games? We focus on depicted laborers. Unlike some manual labor positions, bartenders and mixologists are relatively low-income earners, making an average of $30,000 USD a year (Glassdoor, 2022a, 2022b).

By focusing on precarity, we can examine how video games position labor in relation to livelihood, where a “work to live” mentality is either made visible or invisible in a game's mechanics and narrative. Precarity is constructed through “income instability, lack of a safety net, an erratic work schedule, uncertainty about continuing employment, the blurring of work and nonwork time, and the absence of collective representation” (de Peuter, 2011, p. 419). When money is scarce, precarity is just around the corner. Within the media, poverty and precarity are consistently framed as an individual's fault where appropriate merit and worth can help them escape its clutches and climb to success (Streib et al., 2017). Film, media, and, as we show, games, continue to portray the “American Dream”, where working hard can help get people to the top of the economic ladder of success (Lemke, 2016). In games, meritocracy, which emulates this American Dream, typically drives player progression. Paul’s (2018) exploration of meritocracy shows how it incurred negative community behaviors, however, we turn to its ability to obscure social class precarity that NPCs and player characters experience. Today the notion of the American Dream is transitioning to myth (Mayfield, 2020; Stiglitz, 2012), yet games struggle to express this decline even when they focus on labor.

Just as the “American Dream” argues that economic stability and success come through hard work, games tend to position player agency and success as central mechanics of play. User agency allows players to take on or aid NPCs in their tasks while hopefully succeeding at a game's core goal. As Deery and Press (2017) note, media structures influence class ideology through their inclusion or exclusion of specific class positions. By providing agency in these spaces, games can further reinforce these ideals in their ability to present and obfuscate specific ideologies around labor and class positions. Our goal is not to argue for a game's effectiveness in this but recognize that games offer distinct forms of agency for players to engage with class and labor representations. Games have historically shied away from topics of social class, poverty, and precarity (Lantorno et al., 2021). By recognizing that these themes are rarely explored in tangible ways we ask: what changes when precarity, social class position, and working-class labor are placed at the forefront of play and analysis? We employ bartending and mixology as a case study to ask how play, poverty, and precarity become interrelated within drink making and serving games?

### Bartenders, Mixologists, and Creative Labor

We focus on drink making and serving because the romanticism and cultural import of the role influenced titles specifically focused on these acts. In games, bartending and mixology rely on labor as a core part of narrative design and play. While we focus on their gamed construction, these positions have primarily been embraced in research outside of games.

For clarity, this paper will employ the phrase “drink making and serving” to reference the similarities in labor for bartenders and mixologists. We believe this recognizes the agentic labor practices games afford. Drink making involves meeting the needs of the customers through drink construction. It asks players to create the specific drink a customer wants, to combine recipe ingredients, or pour from a tap. It can be focused on presentation and efficiency. Drink serving, however, prioritizes meeting customer demand and satisfaction through distribution. It asks players to manage a set of “rowdy” customers and get them their orders on time. Success equates “good service” and sets the overall mood for the space. By employing the phrase *drink making and serving,* we focus on games that allow players to both construct *and* serve. In doing so, this exposes a contrast in how bartender and mixologist perform the labor.

Bartenders and mixologists both revolve around beverage concoction and distribution. They are focused on constructing interpersonal exchange between worker and customer (Cowen et al., 1981), but vary in their allowance for craft creativity. The bartender is a drink maker, pouring drinks from a tap or simple menu and serving them to customers. They do not require creative expression in their drink making and their job is focused on quantity over quality, where meeting demand is the bottom line for success. On the other hand, the mixologist is an artisan or culturally knowledgeable tastemaker who adds value and shapes products to consumers’ desires (Ocejo, 2012), A mixologist is perceived as creative, making the drink not just for money but to curate an experience for the customer.

Succinctly, the bartender is a laborer while the mixologist is a creative laborer. Both roles establish relationships with patrons, but the mixologist is differentiated for their skill in crafting a drink. For Ocejo (2010), this labor makes the mixologist a cultural intermediary through craft production, where they redefine the service aspect of their work through their job as experience curator. However, this aspect of the job further hides the economic precarity of their practice Gill and Pratt (2013). argue that the push for creative labor has not removed the precarity of cultural work, where aspirations for success often serve to hide the challenges of the job. Across industries, creative and cultural laborers struggle with precarity even if the “coolness” of their work makes it appear invisible (Murray & Gollmitzer, 2012). Mixologists are no exception to this rule, where the high-brow status of their practice and establishments are not always met with fair pay (Frenette & Ocejo, 2018). This is readily seen in the average salary of a mixologist compared to a bartender where, according to Glassdoor (2022a), bartenders actually make slightly more (33,000 USD) than mixologists (30,000 USD) on average each year. Nevertheless, bartenders and mixologists are both service workers, and their “craft” does not save them from economic insecurity (Frazer, 2017).

The transition from worker to artisan merely reframes the action. The romanticization of a mixologist's labor, or the “coolness” of their jobs, blurs the precarity behind their work (Frenette & Ocejo, 2018). We explore how these assumptions of creativity and precarity transition into game worlds and ask how the role and genre of a game reinforces or pulls away from this discourse. Studying bartenders and mixologists in games allows us to question how the escapism and reality-adjacent space that games create afford differences in interpretation of these roles. If the coolness of the mixologist's job surfaces in these game worlds, does precarity follow and how?

### Past work on Drinking Games

Drink making is a form of crafting, which is a longstanding element of many games. For survival games like, *Minecraft (2011)*, crafting is core to gameplay and research has discussed how this practice is both productive in game and educational for the player (Nebel et al., 2022). Crafting practices ask players to connect their knowledge with game literacies where crafting mechanics are an abstraction of out-of-game concepts into a playable space (Thompson, 2020). We can understand crafting, whether it is a sword, set of gear, or an alcoholic drink as combining assumptions about labor, cultural, and player knowledge into the game. Figuring out which objects combine to produce what is a mix of player's personal experiences and the Gameworld as crafted (Thompson, 2020). As Sullivan et al. (2020) note, labor is fundamental to this process but also inscribes a very productive understanding of work built on gender norms. They look at the massive craft economy of World of Warcraft (2004) to demonstrate how the creative expression is lost in the almost industrialization of object creation. Drink making and serving is a subset of these crafting systems, one that surprisingly escapes *WoW's* robust system, and has grown into a niche of games. By focusing on them through a lens of labor and class we can study the precarity of these depicted jobs. The crafting and distribution of alcohol afford a powerful play space that emulates labor and hints at individual tasks within the establishment of a bar or club.

There is a handful of research that has explicitly looked at the relationship between video games and drinking, but drink making and serving are relatively under-researched. Most work focuses on the interplay between alcohol consumption and play spaces beyond the screen. For example, Sotamaa and Stenros (2019) explore drinking culture and the games within it, while Ream et al., 2011 consider the impacts of drinking on video game play. However, none of this research has translated to drink making processes in video games or the larger history of video game crafting. Instead, researcher attention has been drawn to drink consumption and the games around it. Some work has considered the social spaces created through video games, where digital taverns and social worlds have been expressed as virtual third places allowing users to come together (Moore et al., 2009; Rosenbaum, 2015). However, even these discourses prioritize the culture of community rather than take a lens explicit to labor. In this regard, our analysis of drink making and serving within video games is relatively novel. Our focus on labor, creativity, and precarity shifts discussion from the culture of consumers toward the simulation of labor through the playable drink making and serving process.

## Method

### Game Choice

We began by selecting and categorizing games based on their presentation of bartenders or mixologists. We conducted a series of keyword searches in the Steam store and internet browsers to curate an initial list of 37 games that varied in genre, system requirements, release dates, and stage of completion (i.e., in-development games or game demos). Games were included if they: represented both drink making and serving, primarily engaged with a site of labor such as a bar or club and included labor itself as a core play mechanic. This meant that games, where drink making and serving was in the background or only in a handful of scenes, were not included in the sample.

Since our research prioritized the relationship between labor and precarity, this list narrowed considerably. Some, such as idle clicker games, were removed because they offered little mechanical, visual, or narrative elements to warrant a deeper analysis. Others were not able to be run given our system requirements^1^. Finally, some were ignored because they were only betas or demos of games set to release in the next year or two. This left us with a smaller list which we narrowed down based on a secondary set of criteria. First, we wanted a somewhat historical exploration of the themes starting with earlier games like *Tapper* toward more recent titles. Second, we wanted games that could be placed in comparison to one another by sharing similar play mechanics but a variance in their focus on either a bartender or mixologist. This led to the final choice of *Tapper* to be compared with the browser game *Bartender the Right Mix* (referred to hereafter as *BtRM*), and *VA-11 Hall-A* (pronounced “Valhalla” and referred to hereafter as *VH*) we compare to *Red Strings Club* (referred to hereafter as *RSC*).

Next, we drafted an analysis connecting Consalvo and Dutton's Methodological toolkit with Fernandez-Vara's discussion of game content analysis Consalvo and Dutton’s (2006). toolkit suggests a focus on four play aspects; object inventory, interface study, interaction map, and gameplay logs and each researcher played and recorded their findings in a set of notes. These were consolidated with Fernandez-Vara’s (2019) discussion of content analysis that emphasizes the need to comprehend the formal qualities of a game and research approach, which in our case was labor. Rather than analyze the games as singular entities, we evaluated the role of labor in mechanics, the impact of labor and precarity on the character through narrative, the visualization of economic position in the interface, and references to precarity in the objects we could or could not obtain. Near the end of the individual researcher playthroughs, we compared presentations of labor and creative labor in each of these games.

## Results: Game Summaries and Analysis

### Description of Games

#### Tapper

Released in 1983, *Tapper* is a fast-paced bartending arcade game that involves meeting the rush of beer-thirsty patrons. Both Tapper and their patrons are dressed in cartoonish and culturally stereotypical garb of a saloon-style bartender and saloon-goers which is set against a relatively simple bar space. The bar consists of four horizontal lanes made of long wooden bars (which are reskinned in later levels). At the end of each is a beer tap which players use to quickly fill up a drink and slide it down the bar to satiate the slowly advancing patrons. Gameplay focuses on jumping between bars to slide drinks to patrons and pick up used glasses or leftover tips. As patrons move down the bar to be served, they become increasingly upset until they receive a drink. Otherwise, if they reach the end, Tapper is thrown out.

**Figure 1.**
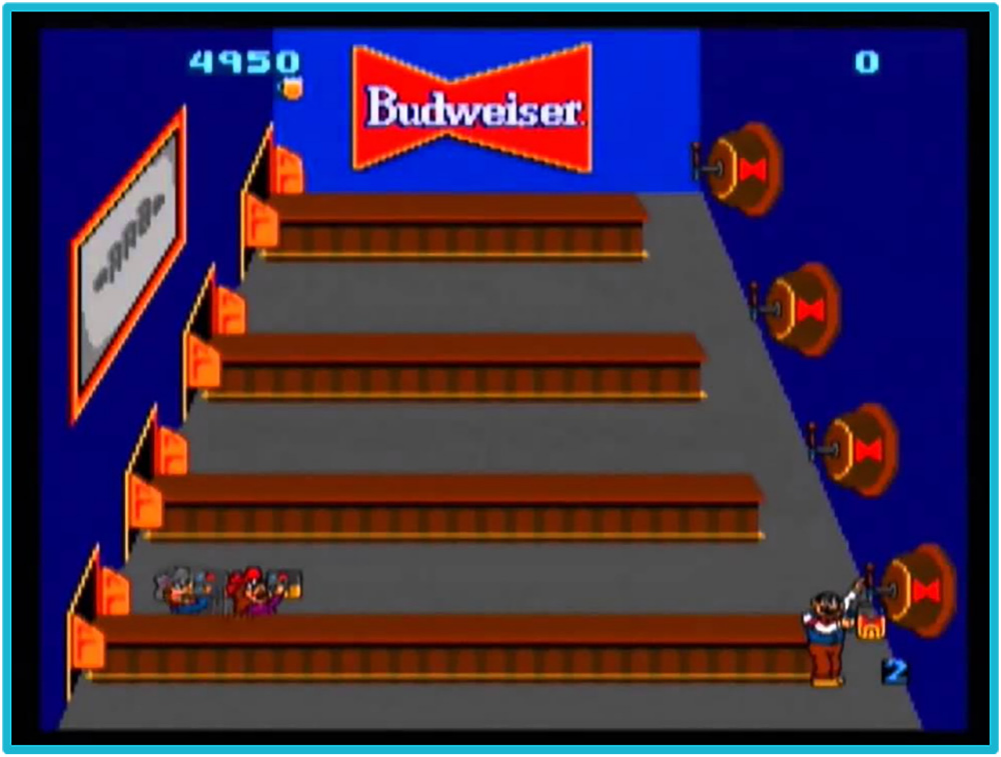
Screenshot of *Tapper* level one interface which starts in a sponsored bar (Midway Games, 1983).

The game's interface is simple (Figure 1), with a score number in the top left with “life” indicators below it and a level marker in the bottom right. As players progress through levels, the scene changes, such as morphing into a sports stadium where the drink on tap becomes replaced with beer carts. Tapper collection of tips and serving of patrons garners points, making a high score indicative of compensation for labor. Similar to crafting to survive in games like *Minecraft*, crafting keeps you in the game. If you work hard, you get points, and you keep your job. A job that becomes increasingly more demanding as you level up, until eventually the patrons forcibly remove you.

The alcohol is always the same, most patrons do not leave tips, and Tapper is trapped in their job until they fail. As a bartender, Tapper's job is to survive an onslaught of repetitive tasks before getting canned, a survival style of play reminiscent of many arcade games.

*Tapper* is considered the first drink making and serving game and an early example of an “advergame”. Shortly after the game's release, the Budweiser company logo replaced the bar's back wall. Interestingly, it is argued that this association was used less to promote Budweiser but instead work with Budweiser's distribution network to get the game into bars (Nelson, 2016). Mark Nelson's analysis of the game's patent discloses that it was initially intended to be played in bars as a game reminiscent of the space, until its popularity saw it in the arcades. The patent described the game's narrative as a, “Video game in which a host image repels ravenous images by serving filled vessels” (Nelson, 2016, p. 2). The core aspect of narrative is the serving of drinks not the drink making. This play mechanic combines what Nelson refers to as, “an abstract order-fulfilment game mapped onto shooter-like concrete mechanics” (p. 6). It contains the demand and serving space of an order fulfillment game, but the rapid fire and endless barrage of enemies found in a shooter. This emphasizes how Tapper is a bartender rather than mixologists, where customers are “ravenous images” that must be satiated by making and serving drinks. This leaves little room for creativity or social discourse between Tapper and the patrons.

#### Bartender the Right Mix

A browser flash game, *BtRM* asks players to impress snooty bartender Miguel with the perfect drink. Players select from a variety of alcohols for Miguel to pour, shake, and eventually serve. Since the goal is to assuage his palette, Miguel delicately tastes each player's drink and has a slapstick reaction based on the quality of the product. This can range from him vomiting, dying, crying, getting hit by a weight, or having starry eyes—should the drink be so good. No matter his reaction, players are presented with a final score alongside the prompt: “Think you can do better?” Unlike the explicit high scores in *Tapper*'s arcade model, *BtRM* asks players to beat personal bests and impress Miguel to win the game. At this point in the game's history, fans have determined which “mix” will get each of Miguel's reactions and subsequent score, making the game relatively “solved”.

**Figure 2.**
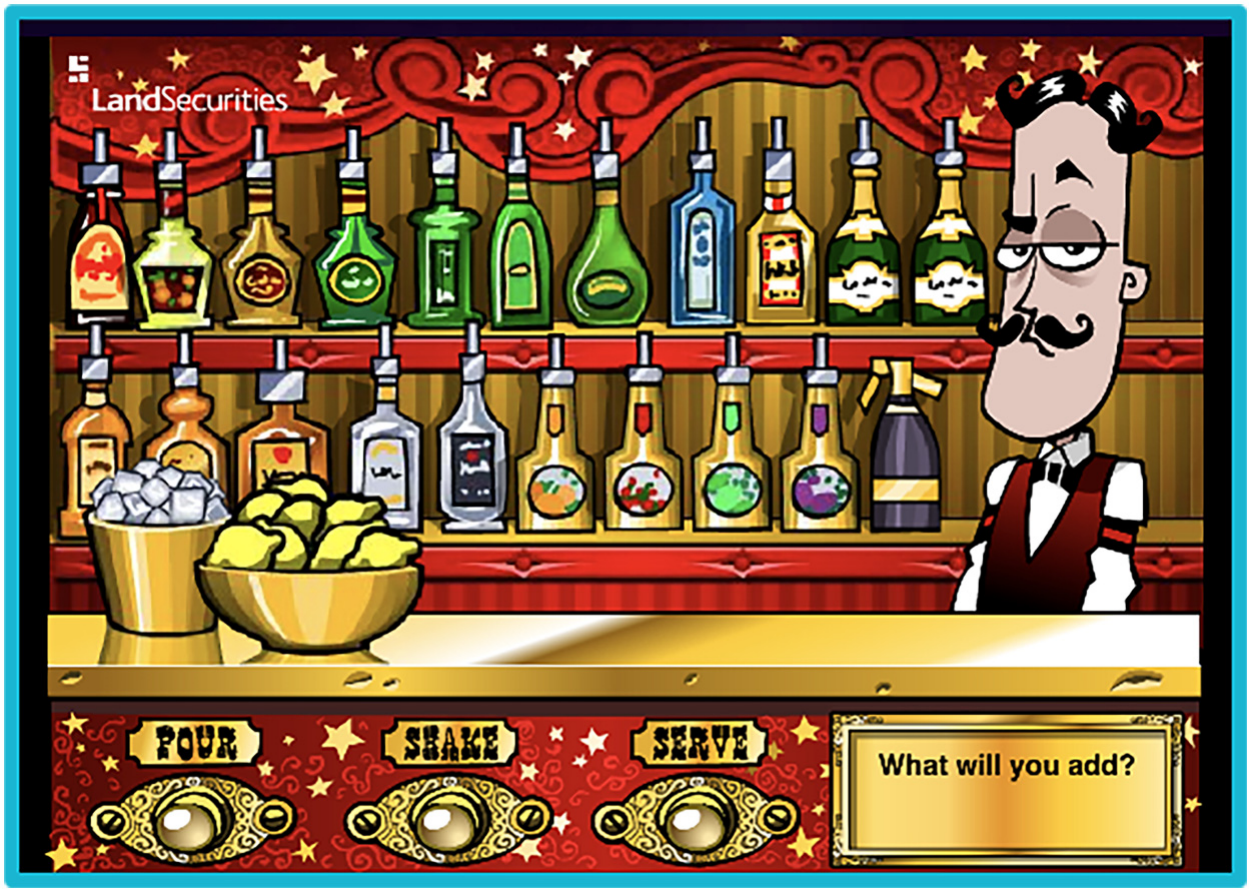
The main player interface for Bartender the Right Mix. From here players select drinks to combine for Miguel (Liquid Light, 2005).

The narrative, visible in the game's interface and title, is that drink making is a difficult, high-skill job, requiring distinct knowledge and abilities to complete. Miguel is the expert, and the gold adorned bar counter gestures toward something higher brow than the wooden bar we find in games like *Tapper* (Figure 2). The desire to appease Miguel's tastes insinuates to players that not just anyone can make the right drink, and the slapstick reactions further solidify the importance of creating the correct mix. Here, labor becomes artisanal, where players transition from unknowledgeable and unsuccessful to a creative expert who can make the “perfect” drink. The game is focused on success just as much as experience, where different guides and playthroughs discuss the vast combinations that can be used to coax specific reactions and experiences from Miguel. Unlike *Tapper,* the pace of the game is neither fast nor demanding; rather, players can take their time as they curate the ideal experience for Miguel through beverage construction. Miguel is both audience and established mixologist, and players ease into the role of mixologist by trial and error or online guides. Players can succeed by crafting the right drink in or out of the game through extensive knowledge of which combinations create which reactionary outputs.

However, no customer ever tastes a drink and money does not exist. Despite the high-brow esthetics, there is no indication that the player or Miguel is getting paid. The game reaffirms the role of drink makers as craft laborers but offers no reward (beyond the prestige of earning a high score) upon assuming the role. Being successful only creates a reaction and does not even guarantee the player a job.

#### VA-11 Hall-A

As its title suggests, *VH* (Sukeban Games, 2016) is a quirky take on drink making and serving set in a cyberpunk city. Players play as Jill, a jaded bartender who quit college due to insurmountable pressure and fell into service work with no inspiration to work elsewhere. Making drinks is done by following a recipe booklet and listening to vague instructions from the clientele. Throughout the game, Jill talks with various characters including humans, androids, and talking dogs. Drink making has an impact on how the game ends and these customers further narrativize the plot through their conversations with Jill and other patrons.

**Figure 3.**
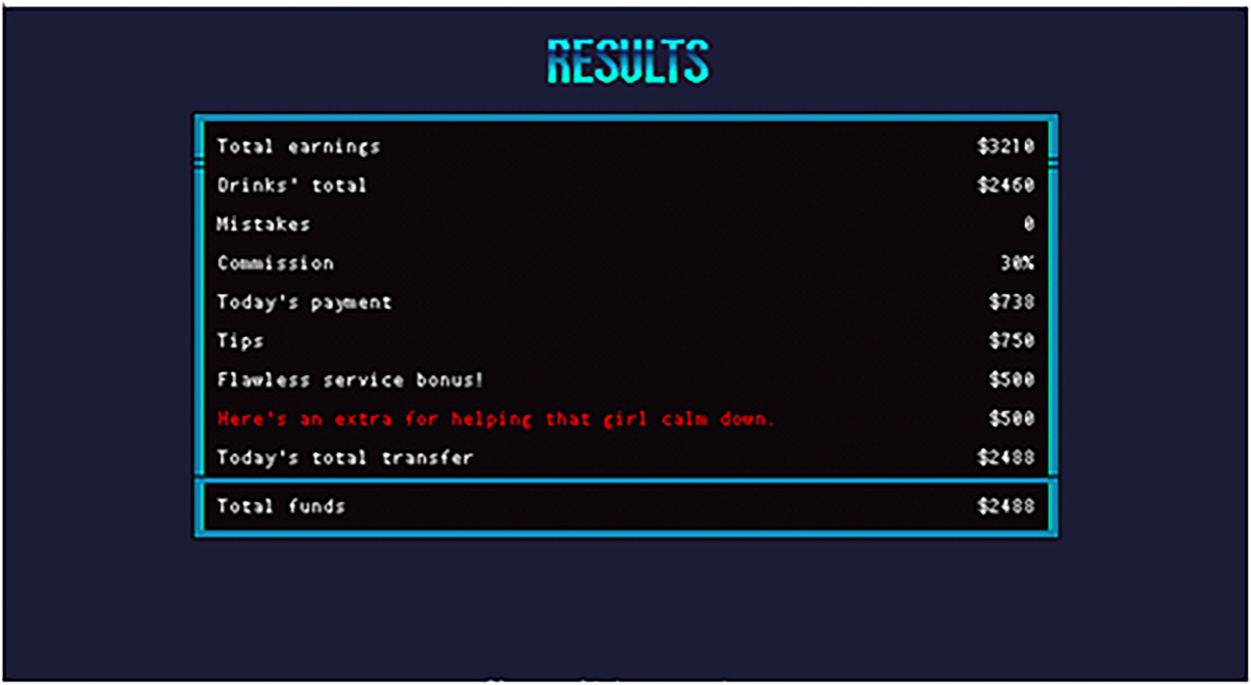
Jill's end-of-shift income broken down to players (Sukeban Games, 2016).

The game oscillates between Jill's shift at the bar and her small one-bedroom studio in the city. Outside of work, players can browse the city's news, visit social media sites, and purchase objects to keep Jill happy and focused. At the end of each shift, Jill gets a breakdown of how much she earned based on what the bar made (Figure 3), and while players are encouraged to spend it on bedroom furnishings (such as posters, music players, or recoloring the space) they need to keep in mind the rent due at the month's end. If Jill's financial situation is not dire and players can buy the objects she wants, a prompt will encourage players to buy things Jill likes. For example, “Jill is lost in thought about a Holo-Plant. Buying it will prevent her from getting too distracted” (Sukeban Games, 2016). If players make this purchase, she is more “focused” at work, and provides player hints on which drinks to make for trickier customers who want to be “surprised” (allowing players a shot at getting every drink order perfect in one playthrough).

Spending and saving money plays into Jill's precarity. Throughout the game, her boss and other supervisory positions mention the impending closure of their bar. While no final directive is given, Jill lives in a state of perpetual precarity where her place of employment, and sole form of revenue, could be lost. This stress is not used to inform long-term saving, rather, the game continually reminds players of the month's rent and the importance of buying commodities to relieve the stress Jill builds up through her work. This positions Jill's financials as something short term where the ability to save for a future is relatively unaffordable. As the game ends, this problem is reflected by several possible endings, one of which involves Jill being kicked out of her apartment.

**Figure 4.**
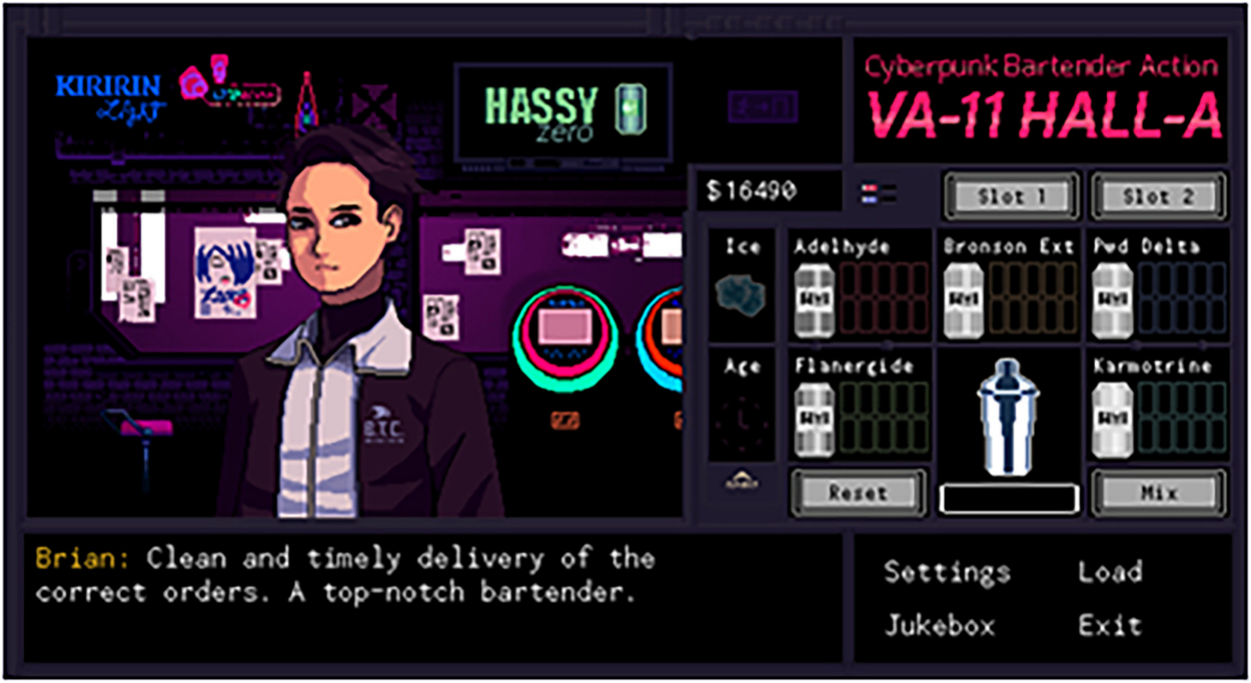
A screen shot of the interface after a successful drink order in *Cyberpunk Bartender Action: VA-11 HALL-A* (Sukeban Games, 2016).

Mechanically, drink making follows a click and drag system. First, players scroll through a menu to find a recipe based on a patrons’ request. They then select and drag alcohol icons to pour the right amounts, click a button to shake the drink or add ice, and then click again to serve it (Figure 4). As employees of the space player agency are restricted to this crafting and the ability to alter the ambiance by selecting a playlist from the bar's sound system. All of this is for customer satisfaction. From the drinks being made and the conversation cues being followed, players have to stay alert in order to get the ending they want. These endings are based on how much Jill's bartending matched the drinks each patron requested and how financially stable she is.

#### Red Strings Club

Developed after *VH* but falling into this, perhaps, emergent cyberpunk drink making sub-genre, *RSC* has players uncover and foil the nefarious actions of megacorporation Supercontinent Ltd. While players jump between three different characters, the majority of their time is spent as the mixologist (named Donovan) for the Red Strings Club, a -way bar where drinks are said to “touch on customers’ emotions.” The player is not a bartender but rather a “muse” or creative spirit that guides Donovan's drink making process. They are called upon when the bartender makes drinks to take control of their hands and determine what to pour. By reading people's emotions, players can pour drinks to suit their tastes and alter the bartender–patron conversation in order to gain more information about the corporate scandal.

Currency in *RSC* is replaced with information. Players never keep track of a wallet, but the game records and documents narrative trajectories and world information which is typically provided by customers in exchange for a drink. Depending on the beverage, patrons will have specific emotional responses and alter how talkative they are about certain questions when pressed. This means that gameplay decisions focus on crafting a drink to manipulate conversation toward the player's narrative goal.

**Figure 5.**
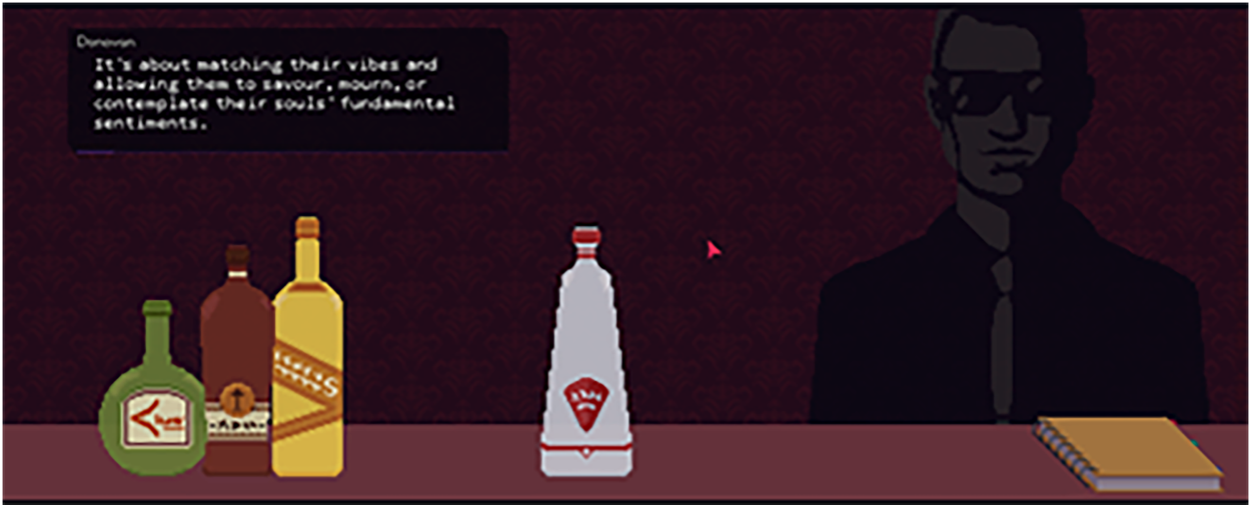
Image of drink making interface. Text is from mixologist Donovan explaining how to mix the perfect drink in Red Strings Club (Devolver Digital, 2018).

Mechanically, unlike the simple clicking and dragging in *VH,* players will rotate their mouse to simulate drink pouring (Figure 5). Players will drag over an alcohol bottle, and gently twist the mouse to control the flow of alcohol toward a glass. As they pour, a small circle indicator moves along the portrait of a customer to help players place it in line with a specific emotion that is being expressed. Depending on the alcohol selected, the indicator will move in a distinct direction, emblematic of making a drink to one's particular tastes. Shaking the drink through vigorous mouse movement and adding ice with a simple click and drop allows players to meet an emotion and serve the drink, bringing them into a new dialogue sequence. Together, this makes drink making the primary narrative motivator and core play mechanic. Beyond this, players will select dialogue boxes to navigate the conversation. As the play progresses, the game provides a visual map of the choices and information players have uncovered to show how those decisions have come to impact the game ending.

## Discussion

### A Difference in Style

Between these games, the distinction of bartender and mixologist presents itself in the game's esthetic. While narrative games like *VH* and *RSC* reflect the variance in the larger story tied to player character labor, mechanically simpler games (like *Tapper* and *Bartender the Right Mix)* reaffirm this distinction by making labor the act solely responsible for player progress. In our sample, *Tapper's* bartender (Tapper) is juxtaposed to *BtRM*'s mixologist (Miguel), while *VH* shows the bartender (Jill) in comparison to *RSC*'s mixologist (Donovan).

### Tapper + Bartender the Right Mix

Both *Tapper* and *BtRM* are short and relatively fast paced. While *BtRM* does not contain the same time-sensitive pressure of serving and retrieving drinks, the simple mechanical options and ability to only create one drink makes gameplay take only a matter of minutes. Both games encourage players to achieve a high score but for different goals. In *Tapper* value is tied to meeting customer demand through service, while in *BtRM* value is on a single creation.

The interfaces in these games further reflect this bartender–mixologist disparity. In *Tapper,* the neon bar sign, wooden tables, and stereotypical beer stein all mimic the setup of a working-class pub. The bar is not fancy, there is only one drink on tap, and the customers are barely engaged with one another or the bartender. In later iterations of the game, the Budweiser-sponsored sign further implies that the game was marketed toward working-class players, or at least intended to be played in spaces geared toward working-class patrons. Alcohol preference and brand consumption have relationships to social class positions (Collins, 2016; Lennox et al., 2018), and as a beverage that is typically cheaper and marketed toward a younger demographic, we can infer that Budweiser's audience is comprised of working-class individuals (Weyler, 2000). *Tapper's* relationship with the company further establishes the scene as that of a bar, run by a bartender rather than an artisanal mixologist.

In contrast, *BtRM* has gold encrusted countertops, a variety of drink selections, and a well-dressed bartender waiting to rate a player-crafted drink. The focus lies not in meeting demand, but on creating something worthy of the high-brow space. While the cartoonish visuals reduce “fanciness” to satire, the interface infers that drink making is a high-skill job. Rather than chug a stein, Miguel sips the player's creation, savoring the drink before reacting in an overly dramatic way.

In *Tapper*, players are a bartender serving one type of drink. It is poured and presented the same way to each customer as quickly as possible. In *BtRM* players are, despite the game's name, a mixologist creating an exquisite product to satiate the taste buds of a “master” drink maker. Creativity, which is nonexistent in *Tapper*, is central to meeting Miguel's standards. While both games have comparable point schemes that hint at the value of a player character's labor, the games paint different pictures of the same job: one of the menial laborers and that of the creative laborer. This is accomplished through a variance in visuals and gameplay mechanics, which tell different stories without additional narrativization.

### Red Strings Club + VA-11 Hall-A

Even when the narrative was prominent, like in *RSC* and *VH*, the variance between the bartender and mixologist is apparent. Players in *RSC* are an artisanal “muse” being invoked by the club's owner; they are an ethereal mixologist. They are creating drinks in a bar with no menu, using intuition and creativity to read a patrons’ emotions and produce drinks that match their desires. The game's framing of the player as a muse implies that they are “special” and one of a kind. Drink making is no longer just labor; it is an identity and a creative energy that allows connection with people through craft labor. Comparatively, players in *VH* are bartenders who serve drinks off a menu, collect nightly tips to scrape up money for rent and bills, and pretend to enjoy the conversation and company of patrons to get through the shift and, hopefully, increase their payout at the end of the night. As a struggling dropout, *VH*'s Jill is not anything special, they are not a hero, and their job is not a passion but a self-imposed career choice.

Such differences in how the games present the labor of drink making and serving directly relates to their depictions of class. Both games hail from the cyberpunk genre and refer to tense socioeconomic order outside of the bar/club, but their understandings of the impacts of a classist society on the working class vary. As a bartender for *VH*, every drink has a cost. Players watch their labor turn a profit for the bar and see their salary in relation to their work's revenue (Figure 3). Every dollar holds value for Jill's life and success at work. Precarity is expressed by the looming need to pay rent, and Jill's competing desires to enjoy some things in life. Although players might feel secure with their funds, as both rent and Jill's personal expenses increase a player's ability to keep their apartment becomes a notable challenge (Blamey, 2020). The game argues that this can be avoided by working hard. However, because purchasing certain items increases Jill's chances of doing their job well, failing to spend money on miscellaneous goods may result in players struggling to know which drink to make a patron.

**Figure 6.**
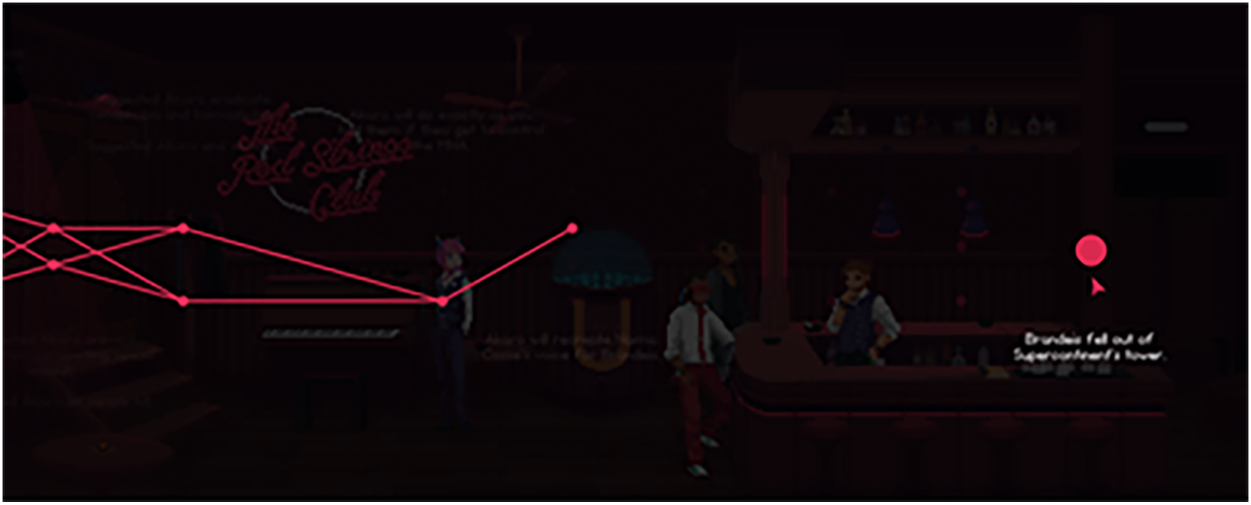
The narrative web that forms in Red Strings Club (Devolver Digital, 2018).

Contrary to *VH*, money is never discussed in *RSC*. Drinks do not hold value, the club does not seem to make any money, and the player never watches their labor create economic value. Just like the drinks, the work of the club is ethereal, meant to meet individual tastes, and produce value by creating a narrative experience that furthers the plot. Despite existing in a world with a tense sociocultural dynamic, player character drink making seemingly exists outside the system. Players are never worried about making ends meet and a sense of individual precarity is never felt. It is replaced with the larger plot of a corporation attempting to manipulate the public's emotional well-being. Emotions and information are the economic system and success is based on what information a player knows and leverages rather than the money they produce and spend. This is visualized through the narrative web that guides the players through the story. As drinks are given to patrons and information is found, the web is filled in, allowing players to see how their labor directly translates to the story's progression (Figure 6).

The variance in the game's mechanics continues to support the bartender mixologist distinction. Scrolling through a menu in *VH* to click and drag drinks compared to physically turning the mouse to pour the beverage in *RSC* club, shows a clear mechanical difference in the two roles. In *VH,* we see the bartender's labor as replaceable, whereas *RSC* relies on skill to pour the right amount into the right glass. For the *VH* bartender, skills are boiled down to choosing the right drink and spending one's money, while the *RSC* mixologist relies on their skill to even make a drink that matches each customer's emotional puzzle.

These differences further translate into how the story is told. In *VH*, the game's ending and success rely on their ability to serve the proper drink and make ends meet. *VH* grounds players to the gritty reality of bartending and economic precarity, where paying rent and determining how you spend your money translates to your overall health and success in the game. In *RSC*, labor produces information where drink making directly fuels conversation about key plot points and future choices. For the mixologist, creative labor is not just that of the drink maker but also that of the story savant. *RSC* shows how creative service labor is valued as something more than economic stability—information.

## Presentation of Social Class

### Creative Labor: Mixologist Versus Bartender

The difference between the creative labor of a mixologist and the drink making and serving of a bartender is felt across all four games. As a bartender labor is rarely creative. Players might have to deduce what patrons are specifically asking for, but the majority of their work is looking up a drink recipe, executing it, and giving it to patrons. The labor mechanics are simplified (clicking a button to serve or sliding it down a bar*)*. *Tapper* is especially emblematic of this job where labor is the sole aspect of the game. Who the customer is, their preferences and desires, are completely removed from *Tapper.* Everyone is the same, everyone is served the same, and anyone can do *Tapper's* job. While *VH's* use of narrative still hints at the cultural value of bartenders in making the third space through conversation, *Tapper* seemingly erases the creative work of developing an atmosphere of experience, dwindling it to simplified mechanics of mute points of agency. Whether it be *Tapper* or *VH,* the bartender is seen as low skill and constantly in fear of losing their job.

As a mixologist, labor is a creative process. Play focuses on artisanal taste and the space of the bar itself. *BtRM* has the bar as the visual forefront of play, and gameplay revolves around the drinks the player chooses to combine. Just as *RSC* asks players to choose and pour the right amount of drinks to match a patron's emotions, *BtRM* focuses on the creation of the drink more so than serving it. In both games, players receive no recipe, just alcohol bottles and a “creative drive.” Here, the mixologist is not held to the pressure of a clock, or making the most money for one's labor, rather the creativity of making drinks is about further telling a story—whether it be the slapstick reaction from Miguel or the continuation of the game's plot. The mixologist is about creative expression.

In these games, creative labor is presented as unconcerned with monetary stresses. Players are situated “above” money as artists or muses whose work has become that of a tastemaker. Just as mixologists are cultural intermediaries to their patrons, so too are these digital mixologists cultural intermediaries to their digital game customers, and the player. Their creative labor not only encourages play, but it offers variance in how the story is told, how relationships come to form, and how the overall play experience is felt by the players. Creative labor is two-fold as players create specific drinks to the taste of the customers and also shape a specific narrative, crafting the game experience to their personal tastes as a player.

Across our sample, creative labor is expressed as something more than simple economic value. Putting a price tag on drink making and serving only works when it is repeatable and doable by anyone. For games that ask for creativity in their drink making and serving (*RSC* and *BtRM*), money disappears, and success relies on patrons’ responses to the player and the product. These games express creative labor as tastemaking and offer no connection to the labor or precarity.

### Poverty and Precarity

Despite the reality of it being a poorly paid profession, poverty and precarity remain somewhat invisible across all our studied titles. *VH* contains the most apt representation of poverty and precarity because its specific game endings, the bar's impending closure, and references to life in a cyberpunk society reaffirm the economic hardship of player character.

*Tapper* and *BtRM* show precarity in how players can lose. For *Tapper,* failing to meet demand and collect tips means they have a lower high score and are eventually removed from their position. In other words, if Tapper cannot keep up they are fired by the customers themselves. In *BtRM*, precarity is witnessed in the reaction of Miguel. Since players are attempting to appease him, failing to impress Miguel, while never explicitly stated, is assumed as a failure in obtaining any permanent status or potential for a job. For both games, precarity is always present in the potential failure to meet customer demand. This is especially poignant in *BtRM,* as there is one correct answer, and players can find it through a simple internet search. In that case, they have cheated precarity and ensured their success, but in doing so the game then ceases to function as a game.

*RSC* avoids representation of the cyberpunk corporate-dystopia's impact on the mixologists’ precarity, only showing class issues through its references to the gameworld's larger lore. As a dystopian state, the game emphasizes that society has been split into factions somewhat based on wealth and knowledge. Since the game replaces money with information, poverty is understood as those with limited information or the ability to use their information. Unlike *VH,* the game never shows the character's lives outside of the bar, but constant reference to the challenges of society suggests that people are struggling to get by economically and brings players into a larger plot of corporate dystopia that has taken over the nation. In the game space, creative labor masks poverty, alongside the larger plot of working-class heroism that asks players to stop nefarious plans. In essence, this “player-muse” position supersedes class and allows the player to rise above the catchall of social ranks, and “save” society. It is not work per se, but creativity.

## Conclusion

This paper has compared how the bartender and the mixologist were presented in four different games that involve drink making and serving to emphasize how game design can make arguments about creative labor and precarity. Whether employing a simple design interface, or elaborate narrative, games can make arguments around the relationship between poverty and labor. As games point to creativity in their work, economic value begins to disappear as other points of value for one's labor begin to surface. These games obfuscate the larger poverty challenges that might exist for these characters, instead appraising creative labor as an artistic practice above bartending. When precarity is included in the narrative, such as in *VH,* games construct it as a point of tension for player decisions that are only used to increase some difficulty in the game, such as making rent versus engaging in retail therapy in VH.

The games discussed in this sample were only a subset of a body of games reflecting the labor of drink making and serving. We narrowed in on these processes as a subset of crafting systems, where our focus on labor demonstrates how precarity becomes translated when shown in creative versus noncreative labor practices. Drink making and serving is one set of crafting that occurs, where the labor practices we discuss could bleed into other craft systems. While we were unable to and refrained from playing all the drink making games in our sample, perhaps other game formats provide insights into the relationships of labor, creativity, and precarity. For instance, idle clicker games could provide a metaphor indicative to the monotonous tasks required by repetitive jobs. Additionally, future work could take our findings of labor in this sub-genre and compare it to the labor embedded in other craft systems. It becomes important that scholarship continues to evaluate representations of class, labor, and precarity within games to evaluate what dimensions are being included and excluded for players.

This analysis has shown how creative labor is embodied in games as a tool to obscure larger discourses of precarity around labor, emphasizing how the translation of labor into gamic spaces alters the severity and relationship to poverty associated with a position. Games reinforce or romanticize the “coolness” of creative labor and in so doing further obfuscate the reality of poverty and precarity that accompany these positions. As we continue to explore the role of social class within video games, we should continue to think about what games are making visible and which pieces remain invisible, playing, analyzing, and challenging these spaces for the dominant ideas they present within the games themselves.

## Notes

1 This was primarily simulation style virtual reality games.
